# Rediscovery of the Microbial World in Microbial Ecology

**DOI:** 10.1264/jsme2.ME2803rh

**Published:** 2013-09-21

**Authors:** Shin Haruta

**Affiliations:** 1 Graduate School of Science and Engineering, Tokyo Metropolitan University, Minami-Osawa 1–1, Hachioji-shi, Tokyo 192–0397, Japan

Recent progress in microbial ecology is allowing for the new discovery of the vast, poorly-characterized microbial world. The following four topics are intensively highlighted as perspectives we can use to look into the novel microbial world in nature; *unexplored habitats*, *unexpected microbial diversity/function*, *microbial community including eukaryotic microbes*, and *complex microbial ecosystems characterized by large data sets*.

## Unexplored habitats

Microorganisms are found in a wide variety of environments and novel microbial species are constantly being reported. Molecular ecological analyses and new efforts to isolate uncultured microbes (40) are opening previously unexplored habitats of microbes. Hydrothermal vents and the deep subseafloor have widely attracted attention of biologists exploring extremophiles (32, 34). Ettoumi *et al*. reported great diversity of marine *Bacillales* in deep-sea sediments (8). Recently, microbes in high-altitude snow (4), in the stratosphere and troposphere (63) and on the International Space Station were investigated (17). The phytosphere is also a collection of interesting habitats for microbes. New findings about microbial communities in the phytosphere of leguminous and other agricultural crops, as well as in natural environments are continuously reported (19, 47, 50, 55, 61). Although metazoans have been well recognized as a habitat of pathogenic microorganisms, we have also become aware of mutualistic relationships between microbes and their host animals (10, 25). Huge numbers of animals and plants remain to be examined as unexplored habitats for microbes.

## Unexpected microbial diversity/function

Analyses based on SSU rRNA genes are a powerful tool for microbial ecological studies. Advances in DNA sequencing techniques deepen our knowledge about bacterial and archaeal worlds (27). Itoh *et al*. utilized colony picking equipment for clone library analysis (21). The equipment used can pick up to 20,000 colonies, providing a high-throughput approach for traditional sequencing analysis. They obtained long DNA sequences of 16S rRNA genes from more than 1,000 clones for 10 libraries each and successfully identified a fine scale succession of bacterial and archaeal communities using this approach.

The physiological properties of microbes are sometimes not supported by phylogenetic relationships. For example, a novel methanogenic lineage was recently found in the class *Thermoplasmata* which had previously consisted of mainly aerobic or sulfur-reducing archaea (6, 18, 39). Analyses of environmental DNAs encoding for physiologically key enzymes involved in processes such as ammonia oxidation, methane oxidation, denitrification, and acetogenesis (29, 46, 58, 62, 65) have discovered functional diversity and patterns of the distribution of microbial communities in nature. Alfreider and Vogt suggested a chemolithoautotrophical bacterial world in groundwater systems by detection of CO_2_-fixation genes (1). Reliable extraction methods of RNA from various environments will accelerate these studies (60). Likewise, stable isotope probing (SIP) of cellular molecules such as nucleic acids and phospholipid fatty acids has been widely utilized for detection of microbes which assimilate particular chemicals (7). Saito *et al*. successfully found novel denitrifying species from rice paddy soil using SIP (45). Microscopic analysis after specific labeling visualizes spatial micro-distribution of microbial cells. Various imaging techniques detecting physiological or metabolic activities have been developed (28, 32, 36) and opened uncovered microbial worlds (44). Micro-environments where microbial communities develop are now able to be characterized using microsensors (48).

Naturally, it should be noted that microorganisms are often in a dormant state in nature. Microbes in a dormant state are not active but are occasionally activated by changes in their environment. Examination of dormant microbes is required for comprehensive understanding of microbial communities, because dormant microbes provide clues to the hidden function of microbial community and they may contribute to stability of microbial ecosystems. Recently, physiological states in response to environmental stress have been well-studied in microbial ecology (16, 22, 67, 69).

Physiological properties which have been determined under pure culture conditions are not always detected in microbial ecosystems. Clarification of the ecophysiology of microbes in microbial ecosystems provides new insights into the microbial world. Both fluctuations of micro-environments and other microbes affect behavior of microbes *in situ. Pseudomonas aeruginosa* is one of the well-studied bacteria in interspecies interactions. Tashiro *et al*. comprehensively reviews the interspecies interaction between *P. aeruginosa* and other bacteria (56). Low molecular-weight compounds such as antibiotics and quorum-sensing signaling molecules secreted by bacteria are widely known to affect growth or transcriptional profile of other bacterial species (56). High molecular-weight compounds such as bacteriolytic enzymes (43) and resuscitation promoting protein (41) also work as intercellular messengers. Clarification of interspecies relationships is uncovering the hidden sides of the microbial world.

## Microbial community including eukaryotic microbes

Prokaryotes, *i.e.*, bacteria and archaea, coexist with eukaryotic microbes such as fungi and protists, including the eukaryotic algae. Molecular ecological studies are being conducted on eukaryotic microflora with useful technical reports being published (23, 49, 52, 64). Diene *et al*. found a novel species of fungi from soils by cultivation and isolation (5). Bao *et al*. reported a pioneering work in which they compared microbial communities including bacteria, fungi and nematodes in soils collected from four sites (2). This report tried to clarify effects of environmental parameters on whole microbial ecosystem in soils using multivariate analyses. They found that soil properties had a simultaneous impact on bacteria, fungi, and nematode communities. Their results also suggested that the nematode community may regulate the bacterial and fungal communities. Steele *et al*. characterized bacteria, archaea and protists in the marine environment after time-series sampling (51). They extracted possible microbial relationships using network analysis based on microbial compositions and environmental parameters.

Viruses should also be considered as a part of microbial ecosystems because prokaryotic communities are known to be affected by phages (54, 66) and eukaryotic microbial communities are also affected by viruses (33).

## Complex microbial ecosystems characterized by large data sets

As introduced above, we have noticed the “complexity” of microbial ecosystems in nature. Multivariate statistics are helpful tools to interpret large data sets, *e.g.*, multidimensional scaling (57), canonical correspondence analysis (37), and principal component analysis (13). A community-wide meta-analysis can also be applied to synthesize data on microbial community composition, microbial processes and environmental parameters (*e.g.*, 3). Characterization of biological processes by microbial communities will be an increasingly important issue for defining ecosystem metabolisms (20, 38, 68). Analysis of community-level responses against disturbance or change in environmental parameters is a practical approach to find out the key members and interspecies relationships within microbial communities (9, 12, 14, 26, 30, 53). At present, several software/web tools are available for data analysis, *e.g.*, ECOMICS for transomics investigation (35) and cMonkey for network modeling of complex systems (42). Likewise, mathematical modeling is also a useful approach to indicate novel interspecies relationships or microbial function in microbial ecosystems (11, 24, 31, 59). In the current issue of Microbes and Environments, advantages and limitations of mathematical modeling are introduced as a mini-review (15). In this review, it is also proposed that theoretical biology/systems microbiology provides a perspective for understanding the plasticity, robustness and stability of complex ecosystems. Theoretical and mathematical approaches combined with traditional microbial ecology are disclosing the complex microbial world and will clarify central and general tenets of microbial ‘societies’ where a variety of microorganisms closely interact with each other.
